# Small bowel melanoma causing obstruction: A case report and a literature review

**DOI:** 10.1016/j.ijscr.2024.109388

**Published:** 2024-02-14

**Authors:** Ammar Mattit, Ibrahim Marrawi, Safouh Kheir, Taha Khamis, Safaa Qatleesh, Muhammad Ali Ousta

**Affiliations:** a Faculty of Medicine, Al-Sham Private University, Damascus, Syria; b Pathology Department, Al-Assad University Hospital, Damascus, Syria; c General Surgery Department, Al-Assad University Hospital, Damascus, Syria

**Keywords:** Primary small bowel melanoma, Melanoma, Intestinal obstruction, Abdominal pain, Case report

## Abstract

**Introduction:**

Primary small bowel melanoma (PSBM) is a rare form of melanoma that originates from the intestinal mucosa. It is typically asymptomatic; however, it can present with non-specific symptoms, which pose challenges in accurately diagnosing the condition. In rare cases, it may manifest as small bowel obstruction, further adding challenges with diagnosis and management.

**Case presentation:**

A 57-year-old male presented to the hospital with complaints of chronic constipation, abdominal pain, and abdominal enlargement. Computed tomography (CT) scan revealed thickening of the jejunum wall, while endoscopy and biopsy revealed nothing. During surgery, surgeons identified and excised a jejunal mass. Subsequent pathological analysis confirmed the diagnosis of melanoma, and post-surgical examination failed to identify primary cutaneous melanoma.

**Discussion:**

PSBM is a rare and aggressive tumor often misdiagnosed due to non-specific symptoms and challenging imaging interpretations. Obstruction and intussusception are uncommon presentations. Surgical resection offers symptom control and improved prognosis, but achieving negative margins can be challenging. Early recognition and diagnosis are crucial for optimal management.

**Conclusion:**

The lack of data in the literature presents challenges in identifying and selecting the optimal approach for managing PSBM. Physicians should increase their awareness of this specific type of tumor to facilitate early-stage diagnosis and provide appropriate care for patients.

## Highlights

• Rare case of primary small bowel melanoma causing intestinal obstruction
• Diagnostic challenges and the need for increased awareness
• Literature review of primary small bowel melanoma cases causing obstruction
• Lack of established guidelines for management and the need for further research

## 1 Introduction

Primary small bowel melanoma (PSBM) originating from the intestinal mucosa is a rare occurrence, with most cases being metastatic tumors. Melanoma is an invasive tumor that spreads locally through lymph nodes and the bloodstream. Although typically asymptomatic, it can present with non-specific and vague symptoms, often leading to late-stage diagnosis [1]. Various diagnostic techniques, such as abdominal ultrasound, barium examination, CT scan, PET scan, gastrointestinal tract endoscopy, and intestinal biopsy, can be utilized; however, the diagnosis remains challenging [2]. Surgical excision with tumor-free margins is the preferred approach for malignant melanoma, but the prognosis remains poor [3]. This report presents a rare case of PSBM causing obstruction. Despite multiple diagnostic methods, a definitive diagnosis was challenging to establish, emphasizing the importance of establishing clear guidelines and standards for managing patients with PSBM. This work has been reported in line with the SCARE criteria [4].

## 2 Case presentation

A 57-year-old male presented to the hospital with complaints of chronic constipation and abdominal pain. The healthcare provider prescribed laxatives as a conservative treatment, which provided temporary relief. However, the patient returned to the hospital after one month with worsening abdominal pain, abdominal enlargement, and unresponsive constipation.

**Fig. 1:**
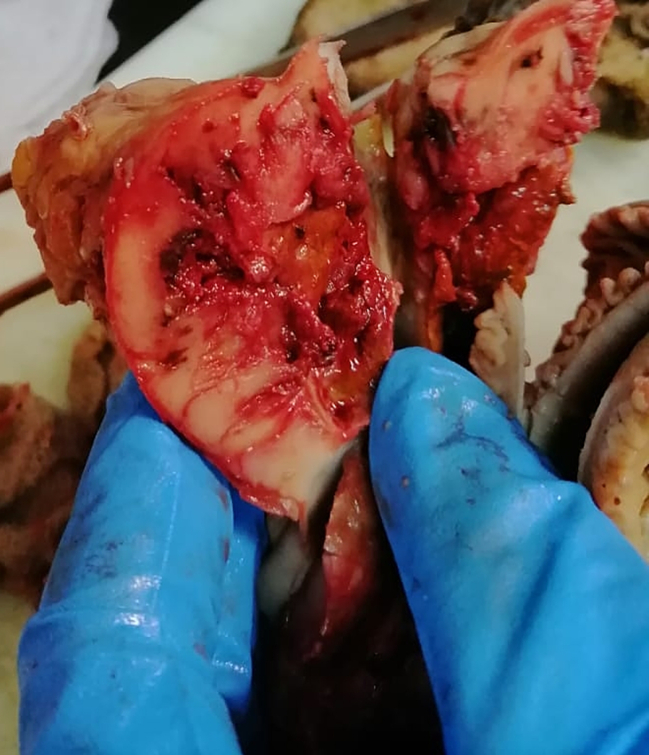
The increased thickness of the jejunal wall.

The CT scan showed thickening of the jejunal wall, measuring 2.7 cm, leading to luminal narrowing and bowel obstruction (Fig. 1), multiple nodal densities were observed in the mesentery, with the largest measuring 36 mm. The CT scan also identified a nodular peripheral mass in the inferior lobe of the right lung, a 2.4 cm mass in the right adrenal gland, and enlarged nodes surrounding the left internal and external iliac artery, as well as the left common femoral artery.

Upper gastrointestinal tract endoscopy revealed erythematous mucosa in the stomach, but no abnormalities were detected upon entering 120 cm into the small intestine. A biopsy of the jejunal wall was inconclusive. Lower gastrointestinal tract endoscopy did not reveal any significant findings.

**Fig. 2:**
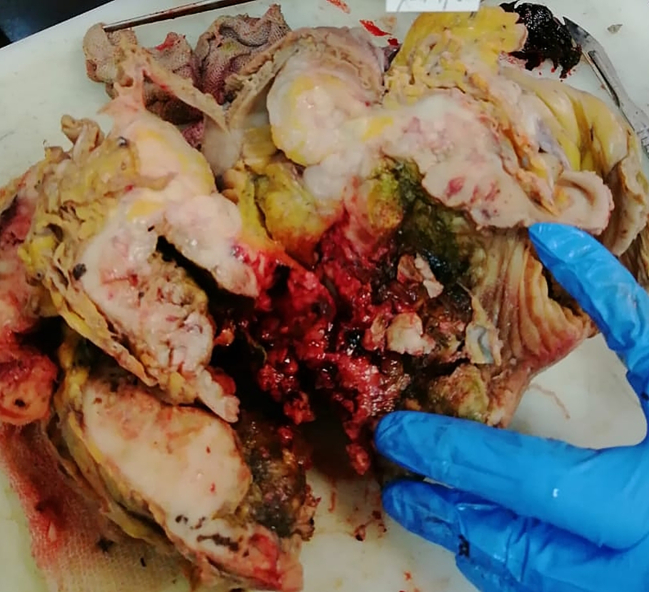
The excised jejunal mass.

Subsequently, laparoscopy was conducted, jejunal mass was identified and a segmental resection of 18.5 cm of the jejunum alongside with its associated mesentery was performed (Fig. 2). Intestinal anastomosis was performed via small lateral incision, prioritizing a less traumatic approach, and a pelvic drainage tube was inserted.

**Fig. 3:**
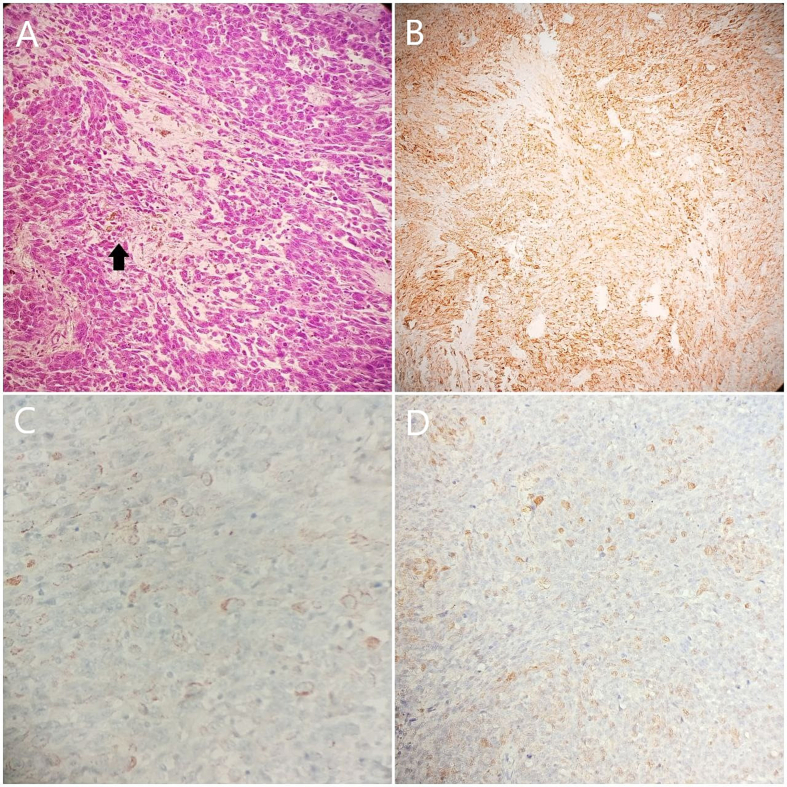
The pathological study; (A) H&E stain: large tumor cells with vesicular nuclei and prominent red nucleoli are observed, surrounded by brownish-black pigment which is melanin (arrow), (B) HMB45 stain: diffuse strong positivity for tumor cells, (C) Melan A stain: patchy strong positivity for tumor cells, (D) S100 stain: focally strong positivity for tumor cells. (For interpretation of the references to colour in this figure legend, the reader is referred to the web version of this article.)

The pathological study of the excised mass confirmed the diagnosis of melanoma based on positive stains for HMB45, S100, and Melan A (Fig. 3). Additionally, there was no evidence of disease involvement in the resected lymph nodes. The patient did not have a past history of skin lesion, and no primary cutaneous melanoma was identified upon post-surgery examination, further supporting the diagnosis of primary small bowel melanoma. However, the presence of the adrenal and the lung masses alongside with the enlarged lymphatic nodes does not definitively exclude the possibility of melanoma of unknown primary. The tumor was clinically determined to be stage IV.

Following the surgery, the patient's medical condition did not allow for additional surgery to address the masses in the lung and adrenal gland. Therefore, chemotherapy was deemed the appropriate course of action. Unfortunately, the patient's condition was deteriorating significantly, and he passed away five months later due to chemotherapy complications.

## 3 Discussion

PSBM is a rare and dangerous tumor that can be easily misdiagnosed. It is important for doctors to be aware of this condition in order to provide appropriate care and reduce mortality. It has a poor prognosis, with a survival rate of less than a year, attributed to its aggressive nature, late-stage presentation, lack of standardized treatment, and subsequent metastasis even with surgical intervention [5].

PSBM is often asymptomatic, but it can manifest with various symptoms such as abdominal pain, weight loss, nausea, vomiting, perforation and chronic iron-deficiency anemia. Obstruction and intussusception are rare occurrences in the context of PSBM, which contributes to the challenge of diagnosing the condition accurately [2]. Gastrointestinal tract melanomas are more commonly diagnosed in older patients, particularly females, and are often at advanced stages upon presentation [5].

Table 1 illustrates all cases of PSBM in the literature that manifest as intestinal obstruction. Among these cases, the majority presented with abdominal pain as the primary symptom. Only one case highlighted anemia as the sole manifestation, posing a diagnostic challenge. Additionally, thickening of the intestinal wall leading to obstruction was observed in only one case, mirroring our own case. It is important to note that the follow-up of most cases did not yield favorable outcomes, highlighting the critical nature of this topic and the need for more research and studies.

**Table 1:** cases of PSBM in the literature that manifest as small bowel obstruction.

Cases	Age	Sex	Clinical presentation	Location	Cause of obstruction	Treatment	Follow-up
[6]	61	Male	Anemia	Jejunum	Intussusception	Surgical resection	NA
[7]	64	Female	Abdominal pain, nausea, vomiting, emesis, melena, weight loss	Proximal jejunum	Intussusception	Surgical resection, and chemo-therapy	Disease-free at 3 years
[8]	64	Male	Abdominal pain, fever, vomiting, constipation	Ileum	Thickened wall	Surgical resection and chemo-therapy	NA
[9]	63	Female	Abdominal pain, vomiting, weight loss	Jejunum	Intussusception	Surgical resection	NA
[10]	78	Male	Abdominal pain, nausea, generalized edema, fever	Distal ileum	Intraluminal mass	Surgical resection	Disease-free at 1 year
[10]	79	Female	Partial obstruction	Distal jejunum	Intraluminal mass	Surgical resection	Died 7 months after surgery
[11]	51	Female	Syncope, melena, malaise, fatigue, abdominal pain, vomiting, weight loss	Small bowel	Intussusception	Surgical resection	NA
[12]	74	Male	Anemia, melena	Distal ileum	Polypoid ileal mass led to intussusception	Surgical resection	Disease-free at 1 year
[13]	42	Female	Abdominal pain, nausea, vomiting,	Ileum	Intussusception	Surgical resection	Died 8 months after surgery
[14]	57	Male	Abdominal pain, bloody stool, nausea, vomiting	ileocecal	Intussusception	Surgical resection	Died 1 month after surgery
[15]	60	Male	Abdominal pain, vomiting, constipation	Ileum	Polypoid ileal mass led to intussusception	Surgical resection	Disease-free at 6 months
[16]	77	Male	Abdominal pain, melena, anemia	Jejunum	Intussusception	Surgical resection	NA
[17]	76	Female	Abdominal pain, fatigue, vomiting, nausea, anorexia, rectal bleeding	Ileum	Intraluminal polyp	Surgical resection	Died 3 months after surgery

Despite the availability of multiple diagnostic methods, including CT scan, PET CT scans, GI endoscopy and intestinal biopsy, interpreting findings in patients with melanoma can be challenging as lesions may exhibit subtle and random distribution, presenting with diverse morphological appearances on imaging and sometimes mimicking other conditions, leading to potential misdiagnosis [18]. In the presented case, multiple diagnostic approaches were employed, but a definitive diagnosis was only established through the surgical approach and subsequent pathological study. Distinguishing between a primary and secondary lesion is also challenging, requiring a comprehensive investigation to exclude the presence of a primary cutaneous lesion [3].

Surgical resection is the preferred treatment approach, as it provides symptom control, better prognosis, and significantly increases survival rates [2]. A study has shown that patients who underwent surgical resection for small bowel melanoma had a median survival of 48.9 months, whereas those who received palliative or nonsurgical treatment had a median survival of only 5.4 months [19]. However, achieving negative margins during resection can be particularly challenging, exacerbated by the presence of lympho-vascular metastasis, which further complicates the surgical procedure [20].

Given the rarity of this disease and the vague symptoms that often lead to misdiagnosis, clinicians should be aware of the importance of recognizing and diagnosing PSBM to facilitate early intervention and treatment planning [3]. Unfortunately, there are currently no established guidelines for the management of mucosal melanomas occurring in the gastrointestinal tract, unlike other types of mucosal melanomas [20].

## 4 Conclusion

In conclusion, PSBM is a rare and aggressive tumor associated with a poor prognosis. The lack of data in the literature and the absence of established guidelines make it challenging for an optimal approach. Moreover, the delayed detection of the tumor creates an additional level of complexity in managing this condition. It is imperative for physicians to enhance their awareness of PSBM, enabling timely and accurate diagnosis, as well as appropriate treatment strategies.

## Consent

Written informed consent was obtained from the patient's wife (the patient passed away) for publication of this case report and accompanying images. A copy of the written consent is available for review by the Editor-in-Chief of this journal on request.

## Ethical approval

Not required for case reports in our hospital. Single case reports are exempt from ethical approval in our institution.

## Funding

The authors declare no source of funding for this manuscript from any organization or any institution.

## Author contribution

AM, IM, SK and TK reviewed the literature and wrote the manuscript. SQ did the pathological study, designed the figures, and participated in the supervision. MAO supervised and reviewed the manuscript. All authors read and approved the final manuscript.

## Guarantor

Dr. Muhammad Ali Ousta.

## Research registration number

Not applicable.

## Conflict of interest statement

All of the authors declared that they have no conflict of interest.
